# Ethyl *trans*-12-(pyridin-4-yl)-9,10-ethano­anthracene-11-carboxyl­ate

**DOI:** 10.1107/S1600536814006588

**Published:** 2014-04-02

**Authors:** S. Chandrasekar, Prakash Sharma Om, V. Srinivasapriyan, M. SureshKumar, C. R. Ramanathan

**Affiliations:** a Centre for Bioinformatics, Pondicherry University, Pondicherry 605 014, India; b Department of Chemistry, Pondicherry University, Pondicherry 605 014, India

## Abstract

In the title compound, C_24_H_21_NO_2_, the residues at the central ethyl­ene bridge are *trans* to each other. The dihedral angles between the pyridine and benzene rings are 67.09 (6) and 61.41 (5)°. In the crystal, centrosymmetrically related mol­ecules are linked into dimers by pairs of C—H⋯O hydrogen bonds.

## Related literature   

For the biological activity of ester derivatives, see: Bi *et al.* (2012 ▶); Bartzatt *et al.* (2004 ▶); Anadu *et al.* (2006 ▶). For conformation studies, see: Nardelli (1983 ▶). For a related structure, see: Gnanamani & Ramanathan (2009 ▶).
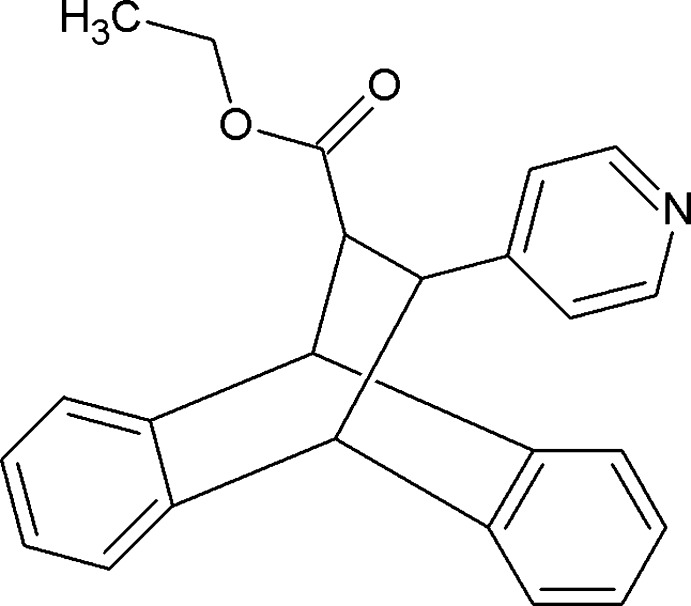


## Experimental   

### 

#### Crystal data  

C_24_H_21_NO_2_
*M* *_r_* = 355.42
Monoclinic, 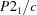

*a* = 10.1733 (19) Å
*b* = 11.156 (2) Å
*c* = 16.361 (3) Å
β = 90.877 (3)°
*V* = 1856.6 (6) Å^3^
*Z* = 4
Mo *K*α radiation
μ = 0.08 mm^−1^
*T* = 298 K
0.40 × 0.38 × 0.20 mm

#### Data collection  

Oxford Diffraction Xcalibur Eos diffractometer
Absorption correction: multi-scan (*CrysAlis PRO*; Oxford Diffraction, 2010 ▶) *T*_min_ = 0.969, *T*_max_ = 0.984
18677 measured reflections
3664 independent reflections
3105 reflections with *I* > 2σ(*I*)
*R*_int_ = 0.027

#### Refinement  

*R*[*F* ^2^ > 2σ(*F* ^2^)] = 0.046
*wR*(*F* ^2^) = 0.118
*S* = 1.05
3664 reflections
245 parameters
H-atom parameters constrained
Δρ_max_ = 0.17 e Å^−3^
Δρ_min_ = −0.25 e Å^−3^

### 

Data collection: *CrysAlis CCD* (Oxford Diffraction, 2010 ▶); cell refinement: *CrysAlis RED* (Oxford Diffraction, 2010 ▶); data reduction: *CrysAlis RED*; program(s) used to solve structure: *SHELXS97* (Sheldrick, 2008 ▶); program(s) used to refine structure: *SHELXL97* (Sheldrick, 2008 ▶); molecular graphics: *ORTEP-3 for Windows* (Farrugia, 2012 ▶); software used to prepare material for publication: *PLATON* (Spek, 2009 ▶).

## Supplementary Material

Crystal structure: contains datablock(s) I, 2R. DOI: 10.1107/S1600536814006588/bt6931sup1.cif

Structure factors: contains datablock(s) I. DOI: 10.1107/S1600536814006588/bt6931Isup2.hkl

CCDC reference: 993524

Additional supporting information:  crystallographic information; 3D view; checkCIF report

## Figures and Tables

**Table 1:** Hydrogen-bond geometry (Å, °)

*D*—H⋯*A*	*D*—H	H⋯*A*	*D*⋯*A*	*D*—H⋯*A*
C18—H18⋯O1^i^	0.93	2.55	3.2612 (18)	134

Symmetry code: (i) 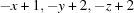.

**Fig. 1.**
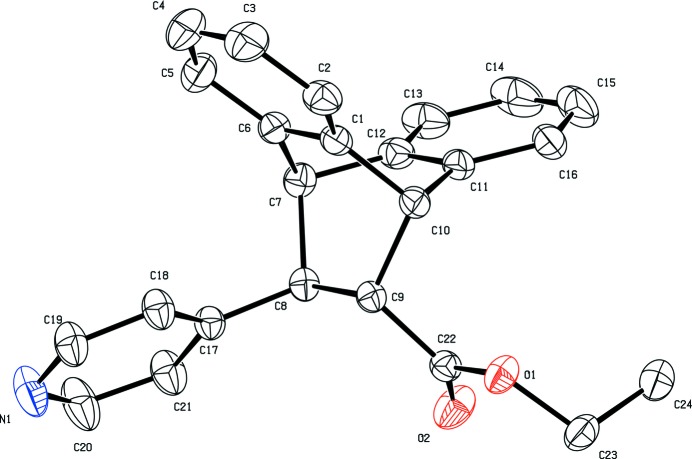
The molecular structure of the title compound, Displacement ellipsoids are drawn at the 30% probability level, H atoms have been omitted for clarity.

**Fig. 2.**
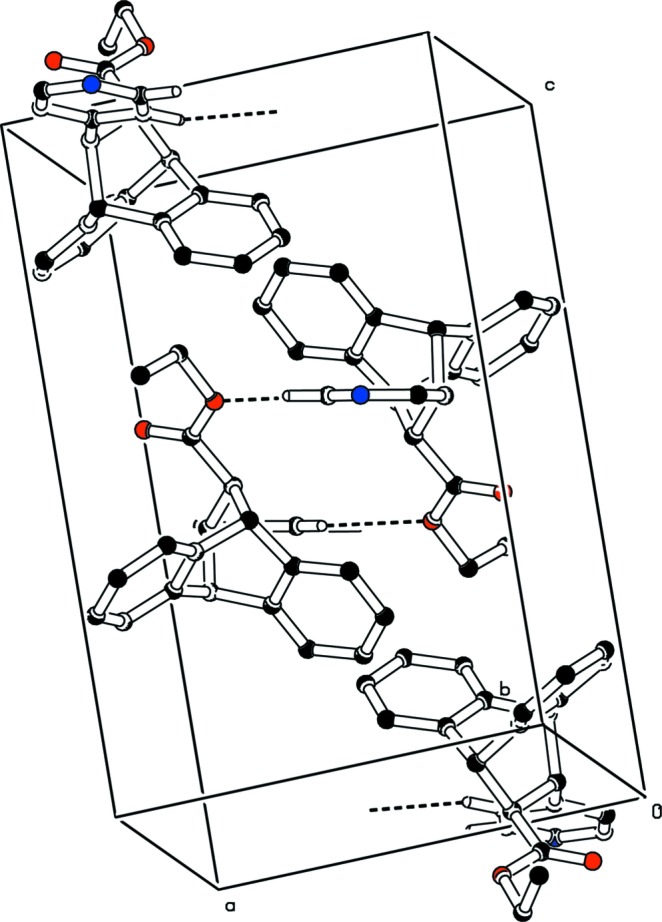
Crystal packing of the title compound, Hydrogen bonds are shown as dashed lines. For the sake of clarity, H atoms not involved in the interactions have been omitted.

## supplementary crystallographic information

### 1. Comment

Ester derivatives of many compounds exhibit a variety of pharmacological properties, for example anticancer, antitumor and antimicrobial activities (Anadu *et al.*, 2006; Bi *et al.*, 2012; Bartzatt *et al.*, 2004). In view of their importance, the title compound was synthesized and we report herein on its crystal structure. In the title molecule (Fig. 1) the fused tricyclic rings [DS (C7) = 0.0051 (1) Å and D2 (C7—C6) = 0.2248 (1) Å], [DS (C7) = 0.0135 (8) Å and D2 (C7—C6) = 0.2358 (6) Å] and [DS (C7) = 0.0126 (9) Å and D2 (C7—C6) = 0.2543 (7) Å] adopt a boat conformation which can be defined by the above asymmetry parameters (Nardelli, 1983). The torsion angles H7—C7—C8—H8 = -65.81 (15)° and H8—C8—C9—H9 = 129.38 (12)°, define the ring fusions involving the fused tricyclic ring system of the ethanoanthracene moeity. The C22—O1 distance [1.326 (2) Å] shows a partial double-bond character and so the C23 maintains planarity with C22, O2 and C9. In the crystal, pairs of centrosymmetrically related molecules are linked into dimers by C18—H18···O1 hydrogen bonds (Fig. 2).

### 2. Experimental

Anthracene (5.34 g, 30 mmol) and 3-(pyridine-4-yl)-acrylic acid ethyl ester (4.4g, 25 mmol) were taken in round bottom flask containing distilled dichloromethane (100 ml). To this mixture anhydrous AlCl_3_ (6.6 g, 50 mmol) was added and stirred at 0 °C for 48 h followed by stirring the reaction mixture at room temperature for 10 h. The obtained dark black solution was poured into water, the organic layer was separated and the aqueous layer was extracted with ether. The crude material was purified through column chromatography using hexane and ethyl acetate in the ratio of 9:1 as eluent. Yield: 5.7 g, (65%).

### 3. Refinement

All H atoms were positioned geometrically, with C–H = 0.93–0.97 Å and constrained to ride on their parent atom,with *U*_iso_(H) =1.5U_eq_(C) for methyl H atoms and 1.2U_eq_(C) for other H atoms.

### Crystal data

**Table:**

C_24_H_21_NO_2_	*F*(000) = 752
*M**_r_* = 355.42	*D*_x_ = 1.272 Mg m^−^^3^
Monoclinic, *P*2_1_/*c*	Mo *K*α radiation, λ = 0.71073 Å
Hall symbol: -P 2ybc	Cell parameters from 9754 reflections
*a* = 10.1733 (19) Å	θ = 2.2–26.0°
*b* = 11.156 (2) Å	µ = 0.08 mm^−^^1^
*c* = 16.361 (3) Å	*T* = 298 K
β = 90.877 (3)°	Block, colourless
*V* = 1856.6 (6) Å^3^	0.40 × 0.38 × 0.20 mm
*Z* = 4	

### Data collection

**Table:**

Oxford Diffraction Xcalibur Eos diffractometer	3664 independent reflections
Radiation source: fine-focus sealed tube	3105 reflections with *I* > 2σ(*I*)
Graphite monochromator	*R*_int_ = 0.027
Detector resolution: 15.9821 pixels mm^-1^	θ_max_ = 26.1°, θ_min_ = 2.0°
ω scans	*h* = −12→12
Absorption correction: multi-scan (*CrysAlis PRO*; Oxford Diffraction, 2010)	*k* = −13→13
*T*_min_ = 0.969, *T*_max_ = 0.984	*l* = −20→20
18677 measured reflections	

### Refinement

**Table:**

Refinement on *F*^2^	Primary atom site location: structure-invariant direct methods
Least-squares matrix: full	Secondary atom site location: difference Fourier map
*R*[*F*^2^ > 2σ(*F*^2^)] = 0.046	Hydrogen site location: inferred from neighbouring sites
*wR*(*F*^2^) = 0.118	H-atom parameters constrained
*S* = 1.05	*w* = 1/[σ^2^(*F*_o_^2^) + (0.0561*P*)^2^ + 0.4341*P*] where *P* = (*F*_o_^2^ + 2*F*_c_^2^)/3
3664 reflections	(Δ/σ)_max_ < 0.001
245 parameters	Δρ_max_ = 0.17 e Å^−^^3^
0 restraints	Δρ_min_ = −0.25 e Å^−^^3^

### Special details

**Table:**

Geometry. All e.s.d.'s (except the e.s.d. in the dihedral angle between two l.s. planes) are estimated using the full covariance matrix. The cell e.s.d.'s are taken into account individually in the estimation of e.s.d.'s in distances, angles and torsion angles; correlations between e.s.d.'s in cell parameters are only used when they are defined by crystal symmetry. An approximate (isotropic) treatment of cell e.s.d.'s is used for estimating e.s.d.'s involving l.s. planes.
Refinement. Refinement of *F*^2^ against ALL reflections. The weighted *R*-factor *wR* and goodness of fit *S* are based on *F*^2^, conventional *R*-factors *R* are based on *F*, with *F* set to zero for negative *F*^2^. The threshold expression of *F*^2^ > σ(*F*^2^) is used only for calculating *R*-factors(gt) *etc*. and is not relevant to the choice of reflections for refinement. *R*-factors based on *F*^2^ are statistically about twice as large as those based on *F*, and *R*- factors based on ALL data will be even larger.

### Fractional atomic coordinates and isotropic or equivalent isotropic displacement parameters (Å^2^)

**Table:**

	*x*	*y*	*z*	*U*_iso_*/*U*_eq_	
C1	0.59145 (13)	0.92171 (12)	0.85231 (8)	0.0392 (3)	
C2	0.46282 (15)	0.90612 (15)	0.82600 (10)	0.0502 (4)	
H2	0.4091	0.8500	0.8510	0.060*	
C3	0.41498 (17)	0.97553 (18)	0.76167 (12)	0.0649 (5)	
H3	0.3289	0.9652	0.7430	0.078*	
C4	0.49392 (19)	1.05918 (18)	0.72543 (11)	0.0685 (5)	
H4	0.4610	1.1050	0.6823	0.082*	
C5	0.62142 (17)	1.07578 (16)	0.75239 (10)	0.0565 (4)	
H5	0.6739	1.1334	0.7280	0.068*	
C6	0.67128 (14)	1.00685 (13)	0.81564 (8)	0.0407 (3)	
C7	0.80801 (13)	1.01204 (13)	0.85311 (9)	0.0406 (3)	
H7	0.8639	1.0697	0.8248	0.049*	
C8	0.79534 (12)	1.04310 (11)	0.94622 (8)	0.0354 (3)	
H8	0.8826	1.0330	0.9715	0.042*	
C9	0.70331 (12)	0.95000 (12)	0.98565 (8)	0.0341 (3)	
H9	0.6244	0.9919	1.0039	0.041*	
C10	0.66015 (13)	0.85398 (12)	0.92048 (8)	0.0381 (3)	
H10	0.6035	0.7923	0.9440	0.046*	
C11	0.78412 (14)	0.80130 (13)	0.88616 (9)	0.0425 (3)	
C12	0.86419 (14)	0.88680 (14)	0.85040 (9)	0.0442 (3)	
C13	0.98283 (16)	0.85354 (18)	0.81716 (11)	0.0623 (5)	
H13	1.0384	0.9106	0.7947	0.075*	
C14	1.0172 (2)	0.7333 (2)	0.81801 (14)	0.0812 (7)	
H14	1.0962	0.7096	0.7951	0.097*	
C15	0.9370 (2)	0.6489 (2)	0.85202 (14)	0.0788 (7)	
H15	0.9615	0.5686	0.8511	0.095*	
C16	0.82018 (18)	0.68175 (15)	0.88763 (11)	0.0582 (5)	
H16	0.7669	0.6247	0.9121	0.070*	
C17	0.75608 (13)	1.17233 (12)	0.95790 (8)	0.0381 (3)	
C18	0.62967 (14)	1.21560 (13)	0.94575 (10)	0.0475 (4)	
H18	0.5619	1.1633	0.9316	0.057*	
C19	0.60451 (16)	1.33606 (14)	0.95456 (11)	0.0568 (4)	
H19	0.5186	1.3623	0.9461	0.068*	
C20	0.81530 (19)	1.37470 (16)	0.98575 (15)	0.0766 (6)	
H20	0.8812	1.4293	0.9993	0.092*	
C21	0.85033 (16)	1.25592 (14)	0.97912 (12)	0.0578 (4)	
H21	0.9368	1.2323	0.9889	0.069*	
C22	0.76667 (13)	0.88949 (12)	1.05943 (8)	0.0371 (3)	
C23	0.73365 (16)	0.73634 (15)	1.15837 (9)	0.0513 (4)	
H23A	0.6605	0.7091	1.1909	0.062*	
H23B	0.7918	0.7833	1.1933	0.062*	
C24	0.8059 (2)	0.63115 (16)	1.12588 (11)	0.0657 (5)	
H24A	0.7502	0.5882	1.0881	0.099*	
H24B	0.8310	0.5792	1.1702	0.099*	
H24C	0.8831	0.6581	1.0983	0.099*	
N1	0.69472 (15)	1.41714 (12)	0.97428 (11)	0.0713 (5)	
O1	0.68494 (10)	0.81027 (9)	1.09130 (6)	0.0474 (3)	
O2	0.87508 (11)	0.90811 (11)	1.08561 (7)	0.0628 (3)	

### Atomic displacement parameters (Å^2^)

**Table:**

	*U*^11^	*U*^22^	*U*^33^	*U*^12^	*U*^13^	*U*^23^
C1	0.0418 (7)	0.0369 (7)	0.0388 (7)	0.0032 (6)	−0.0034 (6)	−0.0068 (6)
C2	0.0442 (8)	0.0539 (9)	0.0522 (9)	0.0000 (7)	−0.0074 (7)	−0.0095 (7)
C3	0.0498 (9)	0.0783 (13)	0.0659 (11)	0.0112 (9)	−0.0219 (8)	−0.0064 (10)
C4	0.0721 (12)	0.0729 (12)	0.0598 (11)	0.0150 (10)	−0.0192 (9)	0.0128 (9)
C5	0.0622 (10)	0.0562 (10)	0.0511 (9)	0.0068 (8)	−0.0028 (8)	0.0117 (8)
C6	0.0442 (8)	0.0404 (7)	0.0374 (7)	0.0065 (6)	−0.0001 (6)	−0.0016 (6)
C7	0.0388 (7)	0.0416 (8)	0.0416 (7)	0.0001 (6)	0.0052 (6)	0.0039 (6)
C8	0.0302 (6)	0.0331 (7)	0.0428 (7)	−0.0015 (5)	−0.0013 (5)	0.0013 (6)
C9	0.0318 (6)	0.0324 (7)	0.0381 (7)	−0.0005 (5)	−0.0001 (5)	−0.0011 (5)
C10	0.0406 (7)	0.0328 (7)	0.0408 (7)	−0.0045 (5)	−0.0042 (6)	−0.0010 (6)
C11	0.0484 (8)	0.0386 (8)	0.0403 (7)	0.0069 (6)	−0.0105 (6)	−0.0065 (6)
C12	0.0421 (8)	0.0510 (9)	0.0393 (7)	0.0092 (6)	−0.0030 (6)	−0.0072 (6)
C13	0.0464 (9)	0.0837 (13)	0.0567 (10)	0.0132 (9)	0.0010 (7)	−0.0188 (9)
C14	0.0606 (11)	0.1010 (17)	0.0817 (14)	0.0389 (12)	−0.0100 (10)	−0.0405 (13)
C15	0.0844 (14)	0.0631 (12)	0.0880 (14)	0.0378 (11)	−0.0288 (12)	−0.0299 (11)
C16	0.0730 (11)	0.0420 (9)	0.0589 (10)	0.0140 (8)	−0.0235 (8)	−0.0121 (7)
C17	0.0380 (7)	0.0338 (7)	0.0426 (7)	−0.0026 (6)	0.0003 (6)	0.0013 (6)
C18	0.0387 (7)	0.0370 (8)	0.0668 (10)	−0.0028 (6)	−0.0044 (7)	−0.0008 (7)
C19	0.0484 (9)	0.0404 (8)	0.0813 (12)	0.0056 (7)	−0.0079 (8)	−0.0006 (8)
C20	0.0603 (11)	0.0398 (9)	0.1291 (19)	−0.0094 (8)	−0.0204 (11)	−0.0138 (10)
C21	0.0433 (8)	0.0423 (9)	0.0873 (13)	−0.0028 (7)	−0.0129 (8)	−0.0065 (8)
C22	0.0372 (7)	0.0355 (7)	0.0385 (7)	−0.0018 (5)	−0.0013 (5)	−0.0025 (6)
C23	0.0600 (9)	0.0524 (9)	0.0414 (8)	−0.0006 (7)	−0.0028 (7)	0.0126 (7)
C24	0.0885 (13)	0.0532 (10)	0.0555 (10)	0.0116 (9)	0.0022 (9)	0.0108 (8)
N1	0.0656 (10)	0.0358 (7)	0.1119 (14)	0.0022 (7)	−0.0146 (9)	−0.0070 (8)
O1	0.0444 (6)	0.0500 (6)	0.0476 (6)	−0.0064 (5)	−0.0044 (4)	0.0145 (5)
O2	0.0502 (7)	0.0725 (8)	0.0651 (7)	−0.0186 (6)	−0.0210 (5)	0.0209 (6)

### Geometric parameters (Å, º)

**Table:**

C1—C2	1.382 (2)	C13—C14	1.386 (3)
C1—C6	1.392 (2)	C13—H13	0.9300
C1—C10	1.5097 (19)	C14—C15	1.370 (3)
C2—C3	1.389 (2)	C14—H14	0.9300
C2—H2	0.9300	C15—C16	1.381 (3)
C3—C4	1.372 (3)	C15—H15	0.9300
C3—H3	0.9300	C16—H16	0.9300
C4—C5	1.376 (3)	C17—C21	1.378 (2)
C4—H4	0.9300	C17—C18	1.385 (2)
C5—C6	1.380 (2)	C18—C19	1.376 (2)
C5—H5	0.9300	C18—H18	0.9300
C6—C7	1.513 (2)	C19—N1	1.325 (2)
C7—C12	1.510 (2)	C19—H19	0.9300
C7—C8	1.5695 (19)	C20—N1	1.326 (2)
C7—H7	0.9800	C20—C21	1.377 (2)
C8—C17	1.5089 (19)	C20—H20	0.9300
C8—C9	1.5460 (17)	C21—H21	0.9300
C8—H8	0.9800	C22—O2	1.1952 (17)
C9—C22	1.5180 (18)	C22—O1	1.3258 (16)
C9—C10	1.5693 (18)	C23—O1	1.4538 (17)
C9—H9	0.9800	C23—C24	1.487 (2)
C10—C11	1.508 (2)	C23—H23A	0.9700
C10—H10	0.9800	C23—H23B	0.9700
C11—C16	1.383 (2)	C24—H24A	0.9600
C11—C12	1.389 (2)	C24—H24B	0.9600
C12—C13	1.382 (2)	C24—H24C	0.9600
			
C2—C1—C6	120.50 (14)	C13—C12—C7	126.37 (16)
C2—C1—C10	126.33 (13)	C11—C12—C7	113.46 (12)
C6—C1—C10	113.17 (12)	C12—C13—C14	118.5 (2)
C1—C2—C3	119.01 (16)	C12—C13—H13	120.7
C1—C2—H2	120.5	C14—C13—H13	120.7
C3—C2—H2	120.5	C15—C14—C13	121.14 (18)
C4—C3—C2	120.41 (16)	C15—C14—H14	119.4
C4—C3—H3	119.8	C13—C14—H14	119.4
C2—C3—H3	119.8	C14—C15—C16	120.74 (18)
C3—C4—C5	120.54 (16)	C14—C15—H15	119.6
C3—C4—H4	119.7	C16—C15—H15	119.6
C5—C4—H4	119.7	C15—C16—C11	118.55 (19)
C4—C5—C6	119.96 (17)	C15—C16—H16	120.7
C4—C5—H5	120.0	C11—C16—H16	120.7
C6—C5—H5	120.0	C21—C17—C18	116.18 (13)
C5—C6—C1	119.57 (14)	C21—C17—C8	119.60 (13)
C5—C6—C7	127.44 (14)	C18—C17—C8	124.18 (12)
C1—C6—C7	112.98 (12)	C19—C18—C17	119.94 (14)
C12—C7—C6	107.38 (12)	C19—C18—H18	120.0
C12—C7—C8	105.63 (11)	C17—C18—H18	120.0
C6—C7—C8	108.25 (11)	N1—C19—C18	124.26 (15)
C12—C7—H7	111.8	N1—C19—H19	117.9
C6—C7—H7	111.8	C18—C19—H19	117.9
C8—C7—H7	111.8	N1—C20—C21	124.93 (16)
C17—C8—C9	115.20 (11)	N1—C20—H20	117.5
C17—C8—C7	111.08 (11)	C21—C20—H20	117.5
C9—C8—C7	108.42 (10)	C20—C21—C17	119.42 (15)
C17—C8—H8	107.3	C20—C21—H21	120.3
C9—C8—H8	107.3	C17—C21—H21	120.3
C7—C8—H8	107.3	O2—C22—O1	123.76 (13)
C22—C9—C8	112.21 (10)	O2—C22—C9	125.88 (13)
C22—C9—C10	110.35 (11)	O1—C22—C9	110.36 (11)
C8—C9—C10	109.87 (11)	O1—C23—C24	110.02 (13)
C22—C9—H9	108.1	O1—C23—H23A	109.7
C8—C9—H9	108.1	C24—C23—H23A	109.7
C10—C9—H9	108.1	O1—C23—H23B	109.7
C11—C10—C1	107.48 (11)	C24—C23—H23B	109.7
C11—C10—C9	106.99 (11)	H23A—C23—H23B	108.2
C1—C10—C9	106.37 (11)	C23—C24—H24A	109.5
C11—C10—H10	111.9	C23—C24—H24B	109.5
C1—C10—H10	111.9	H24A—C24—H24B	109.5
C9—C10—H10	111.9	C23—C24—H24C	109.5
C16—C11—C12	120.81 (15)	H24A—C24—H24C	109.5
C16—C11—C10	126.33 (15)	H24B—C24—H24C	109.5
C12—C11—C10	112.86 (12)	C19—N1—C20	115.26 (14)
C13—C12—C11	120.17 (15)	C22—O1—C23	117.80 (11)
			
C6—C1—C2—C3	0.9 (2)	C16—C11—C12—C13	−1.3 (2)
C10—C1—C2—C3	−179.47 (14)	C10—C11—C12—C13	178.93 (13)
C1—C2—C3—C4	−0.7 (3)	C16—C11—C12—C7	178.95 (13)
C2—C3—C4—C5	−0.2 (3)	C10—C11—C12—C7	−0.77 (17)
C3—C4—C5—C6	0.9 (3)	C6—C7—C12—C13	126.43 (16)
C4—C5—C6—C1	−0.7 (2)	C8—C7—C12—C13	−118.21 (16)
C4—C5—C6—C7	179.87 (16)	C6—C7—C12—C11	−53.89 (15)
C2—C1—C6—C5	−0.2 (2)	C8—C7—C12—C11	61.47 (14)
C10—C1—C6—C5	−179.89 (13)	C11—C12—C13—C14	2.1 (2)
C2—C1—C6—C7	179.33 (13)	C7—C12—C13—C14	−178.20 (15)
C10—C1—C6—C7	−0.36 (17)	C12—C13—C14—C15	−1.0 (3)
C5—C6—C7—C12	−126.13 (16)	C13—C14—C15—C16	−1.0 (3)
C1—C6—C7—C12	54.38 (15)	C14—C15—C16—C11	1.8 (3)
C5—C6—C7—C8	120.26 (16)	C12—C11—C16—C15	−0.6 (2)
C1—C6—C7—C8	−59.23 (15)	C10—C11—C16—C15	179.04 (15)
C12—C7—C8—C17	172.86 (11)	C9—C8—C17—C21	135.65 (15)
C6—C7—C8—C17	−72.38 (14)	C7—C8—C17—C21	−100.59 (16)
C12—C7—C8—C9	−59.59 (13)	C9—C8—C17—C18	−46.84 (19)
C6—C7—C8—C9	55.17 (14)	C7—C8—C17—C18	76.93 (17)
C17—C8—C9—C22	−109.03 (13)	C21—C17—C18—C19	0.4 (2)
C7—C8—C9—C22	125.81 (12)	C8—C17—C18—C19	−177.20 (15)
C17—C8—C9—C10	127.81 (12)	C17—C18—C19—N1	0.2 (3)
C7—C8—C9—C10	2.65 (14)	N1—C20—C21—C17	1.0 (4)
C2—C1—C10—C11	125.88 (15)	C18—C17—C21—C20	−0.9 (3)
C6—C1—C10—C11	−54.46 (15)	C8—C17—C21—C20	176.79 (17)
C2—C1—C10—C9	−119.80 (15)	C8—C9—C22—O2	−0.6 (2)
C6—C1—C10—C9	59.86 (14)	C10—C9—C22—O2	122.24 (16)
C22—C9—C10—C11	−68.66 (14)	C8—C9—C22—O1	−179.85 (11)
C8—C9—C10—C11	55.59 (14)	C10—C9—C22—O1	−56.96 (14)
C22—C9—C10—C1	176.69 (11)	C18—C19—N1—C20	−0.1 (3)
C8—C9—C10—C1	−59.07 (13)	C21—C20—N1—C19	−0.4 (3)
C1—C10—C11—C16	−124.66 (15)	O2—C22—O1—C23	−4.4 (2)
C9—C10—C11—C16	121.43 (15)	C9—C22—O1—C23	174.85 (12)
C1—C10—C11—C12	55.04 (15)	C24—C23—O1—C22	−83.74 (17)
C9—C10—C11—C12	−58.87 (15)		

### Hydrogen-bond geometry (Å, º)

**Table:**

*D*—H···*A*	*D*—H	H···*A*	*D*···*A*	*D*—H···*A*
C18—H18···O1^i^	0.93	2.55	3.2612 (18)	134

Symmetry code: (i) −*x*+1, −*y*+2, −*z*+2.
